# Abstract of Proceedings of the Buffalo Medical Association

**Published:** 1870-04

**Authors:** Thos. M. Johnson

**Affiliations:** Secretary


ART. III.—*Abstract of Proceedings of the Buffalo Medical Association.*

TUESDAY EVENING, April 5th, 1870.

ANNUAL MEETING.—The meeting was called to order by the President.

Members present—Drs. Miner, Rochester, Diehl, Phelps, Trowbridge, Wetmore, Burger, Whitney, Samo, Gay, Strong and Johnson.

The Treasurer's Report was read, accepted, and referred to the Auditory Board.

The Librarian's Report was read and adopted.

The Secretary's Report was read and adopted.

Election of officers being next in order the following officers were elected for the ensuing year:

S. W. WETMORE, President

THOS. M. JOHNSON, Vice President.

WM. J. PHELPS, Secretary.

C. DIEHL, Treasurer.

JAS. B. SAMO, Librarian.

Dr. J. Trowbridge, of the Committee of Portraits, made a brief report of the labors of the committee, after which, on motion of Dr. Strong, the Committeee was empowered to purchase portraits of deceased members when necessary to do so.

Dr. Rochester moved that the Secretary procure an album containing portraits of all the members of this association. Carried.

On motion of Dr. Rochester, a committee of three was appointed to confer with the trustees of the Grosvenor Library in the selection of medical books for that library. The members of this committee are Drs. Rochester, Strong and Miner.

By vote, the Committee on Portraits was granted further time.

By vote, Dr. Samo was granted further time to prepare his essay.

Dr. Rochester said that in the late trial of Mrs. Carney, for murder, the very important question came up, whether it is possible to tell from the appearance of the wounds found upon a dead body that they were made before or after death, whether tumefaction and ecchymosis will occur if a wound or contusion be made after death. Dr. R. thought the question one of great interest to the profession and mentioned it to hear the opinions of the profession upon the subject.

Dr. Miner said that he had very recently been invited by Coroner Burke to examine, in connection with Dr. Green, a body which had been in the water two or three months, which had marks of severe injury both upon the forehead and upper portion of the head. The question was to determine, if possible, if the wounds were received previous to death; the brother of the subject being under arrest as the murderer. The wounds contained coagulated blood, the parts were discolored for some distance, the bone, even beneath the periosteum, showed discoloration. He had no hesitation in swearing positively and confidently that the injuries were received previous to cessation of circulation—previous to death. Dr. Green was also as positive upon this point. It was well to give the subject full consideration, but it seemed to him that the physicians called in the suit mentioned by Dr. Rochester were correct, and will be sustained by the profession and by any investigations which may be made upon the subject.

Dr. Gay thought the subject of great interest, and believed that in case death occurred suddenly, immediately after the injury there would be no coagula and perhaps no tumefaction. But in case death came on slowly, or at considerable time after the injury, the reverse of these conditions might be found.

Dr. Strong said—Mr. President: It seems to me, in view of recent events in court, that the query suggested by Dr. R. is at once timely and important.

What are the facts in the case referred to? These—viz. and briefly: A healthy boy, of some seven years, was found, after two days search, in a well, constructed of stone, some sixteen feet deep, half full of water. The well was three and one-half feet in diameter, and covered by a plank, in which, directly over the center of the well, was a square opening fourteen inches in diameter.

The testimony of the medical gentlemen (one of them the coroner) who made the *post mortem* examination was to the effect, that upon the head of the child was found three contused wounds, caused by some blunt instrument, one of which was directly above the right eye-brow, and another on the top of the head, 1-2 to 3-4 of an inch in length, and through the integument and muscles down to the cranium but with no fracture of the skull. Around both wounds, for a space of two or three inches in diameter, (I believe,) there was considerable effusion of blood and lymph, with tumefaction.

The theory of the prosecution, and the judgment of the physicians was, that these wounds were inflicted before death for some hour or two, and, the child, either dead or insensible from the injuries, was thrust into the well through the fourteen inch aperture.

The theory of the defense, *sustained too by medical testimony*, (if I am not in error,) that wounds of the character described, might have been *post mortem*, or received at the moment of drowning, by the head striking against the stones of the wall, and by consequence the case was one of accidental drowning.

The question raised is (and it is obviously one of very great importance to have determined, if it is not already determined,) could it be possible that such an amount of coagulation, effusion and tumefaction as was proven might be *post mortem?* Or even an effect from injuries received at the moment of drowning?

The received view, as I have always supposed, is, that effusions, whether of blood, lymph or serum and consequent tumefaction is intrinsically a vital process, necessitating of course the action of the living solids and fluids,—and have nothing in common with *post mortem* changes.

If this view is correct, the idea, that in the case in hand, the wounds could have been received after death is wholly untenable.

The only remaining question is, could their appearances and conditions have had time to occur in the act of drowning? To settle this point involves the consideration of the length of time that elapsed while the child was struggling for its life in the water, assuming that he was conscious--of all which there was nothing proven or positively known. Being hypothetical, of course there may easily be differences of opinion and judgment in the premises.

The medical testimony was that the wounds must have been received an hour or two at least before life was extinct. Of course no *such* time could have elapsed after the child struck the water; and if rendered insensible, by the force causing the wound, as was thought, no appreciable time, certainly no sufficient time to allow of the vital process involved, *could* have passed before death.

It is a matter of every day observation, that effusions of blood and lymph, begin instantly upon the reception of a wound involving rupture or lesion of the vessels. This process is gradually and steadily progressive up to a certain point—varying in rapidity with the seat of injury and other circumstances.

In the case under consideration, the data pertaining to the duration of life, insensibility, etc., were wholly wanting, except inferentially.

But if the examining physicians give accurate statements of the extent and character of the lesions, (as I believe was unquestioned,) it would seem to be difficult, if not impossible to believe, that the injuries were received in the act of drowning—and most certainly not after life was extinct.

And yet, the accused in this case, was acquitted upon the theory, if I am not mistaken, that the wounds *were* so produced.

In view of these facts, I repeat, Mr. President, it is not only timely, but vastly important, that the point at issue should be calmly considered, with the view, that, upon the physiological and pathological laws which obtain in the case, medical testimony may be a unit—and the ends of justice thus be subserved.

Dr. Wetmore said he had never seen signs of tumefaction after death, but had often seen depression at the point of a blow. Have seen contusions made after death that would show as in life but in less degree. If the scalp is bruised after death it will dry and stick to the skull. Blood will not coagulate after death. Have seen ecchymosed spots produced by blows upon the dead body.

Roseola, whooping cough and mumps were reported as prevailing.

Dr. Diehl was elected to read an essay at some future meeting of the association.

Adjourned. THOS. M. JOHNSON,
SECRETARY.

